# Solid Conical Cap-closing Hollow Tube Growth by Axial Screw Dislocations

**DOI:** 10.1038/s41598-017-03109-w

**Published:** 2017-06-05

**Authors:** Yanhui Chu, Jinjin Li, Jikun Chen

**Affiliations:** 1 School of Materials Science and Engineering, South China University of Technology, Guangzhou, 510641 China; 2 Department of Micro/Nano-electronics, Shanghai Jiao Tong University, Shanghai, 200240 China; 3 School of material science and engineering, University of Science and Technology of Beijing, Beijing, 100083 China

## Abstract

Exploration of the mechanisms for growth of the nanostructures is the key point to achieve nanomaterial syntheses with precisely controlled morphology and structure. Herein, we reported a new mechanism that realized the growth of solid conical cap-closing hollow tube by axial screw dislocations in the formation α-Al_2_O_3_ nanowires. A hollow tube was firstly grown by axial screw dislocations in the formation α-Al_2_O_3_ nanowires through vapor-phase synthesis. Afterwards, the hollow tube was closed up by generating a solid conical cap with axial screw dislocations based on the competition between the surface energy and the strain energy of screw dislocation controlled by the growth environment. The solid conical cap-closing hollow tube growth model based on the axial screw dislocations is expected to be a general growth mechanism for nanowires within low supersaturation. This study enriches the fundamental understanding with respective to the kinetics of nanostructured crystal growth and provides guidance to the precise structure control in nanosynthesis and manufacturing.

## Introduction

Nanoscience and nanotechnology are important to aspects such as electronic and photonic devices, and/or biosensors^1–4^. How to design the effective and efficient bottom-up synthesis strategies for nanomaterials to realize precise control of their morphology is the crucial significance for both fundamental research and practical applications^5–7^. In particular, the one-dimensional (1D) nanomaterials including nanowires, nanobelts, and nanotubes receive considerable interests for their practical applications. One typical example is the promising physical properties observed in 1D nanomaterials, which is important for nanoelectronics, nanophotonics, solar energy conversion, thermoelectricity, electrochemical energy storage, and chemical and biological sensing^8–10^. Therefore, improving fundamental understanding of the growth of 1D nanomaterials is essential for further developing rational and controllable synthesis to yield nanoscale morphologies suited for specific applications.

The fundamental question with respective to how 1D nanomaterials grow has long fascinated scientists. Up to now, the fundamental mechanisms used to interpret the growth of 1D nanomaterials are layer-by-layer (LBL) and catalyst-mediated LBL, which are associated to approaches such as vapor-solid growth, vapor-liquid-solid growth, vapor-solid-solid growth, solution-liquid-solid growth^11–14^. In addition, the screw dislocation-driven growth mechanism, known as a typical bulk crystal growth model, has been also demonstrated as an interpretation for 1D nanomaterial growth. Resent research highlighted an important role of screw dislocations in the growth of 1D nanomaterials including nanowires, and nanotubes^15–18^. Nevertheless, the increasing demand of great variety of nanoscale morphologies and structures requires further explorations of new mechanisms to better address a substantial portion of phenomena associated to nanocrystal growth. In this paper, a solid conical cap-closing hollow tube growth mechanism by axial screw dislocations in the formation of α-Al_2_O_3_ nanowires is observed. It yields a terraced surface with a dislocation core and spiral pattern on the nanowire top and a solid conical cap-closing hollow tube structure within the nanowires. The proposed solid conical cap-closing hollow tube model is driven by axial screw dislocations along the nanowire length. The structure evolution from hollow tube to solid in the nanowires is because of the competition between the surface energy and the strain energy of the screw dislocation at different growth conditions. Observation and investigation of the solid conical cap-closing hollow tube growth model by axial screw dislocations enrich our understanding of nanoscale crystal growth mechanisms. This is expected to guide precise structure design in nanosynthesis and manufacturing.

## Experimental procedure

Al_2_O_3_ nanowires were synthesized via vapor-solid deposition process on the as-prepared SiC-Si-Al_2_O_3_ ceramics. It has two steps. In the first step, SiC-Si-Al_2_O_3_ ceramics were prepared on the cubic graphites (10 mm × 10 mm × 10 mm) by reactive sintering in the furnace. The precursor powder of pressure-less reactive sintering was mixed as follows: 60–75 wt.% Si, 10–15 wt.% Al_2_O_3_, 7–15 wt.% graphite, and 5–10 wt.% SiC. Details of preparation of SiC-Si-Al_2_O_3_ ceramics on the cubic graphites were similar to that of the reported SiC-Si ceramics^19^. In the second step, the as-prepared SiC-Si-Al_2_O_3_ ceramics were placed at the center of an alumina tube in the furnace (as the precursor and substrates). After evacuating the furnace for 3 times, Argon carrier gas (99.95%, purity) was introduced into the system with the flow rate of 50 sccm, while oxygen (99.95%, purity) with the flow rate of 2 sccm were used as the precursor. The samples were heated from room temperature to 1500 °C at a rate of 10 °C/min and held for 2 h, followed by furnace cooling to room temperature. To adjust the kinetic for the growth of Al_2_O_3_ nanowires, the other flow rates of the oxygen precursor (0.5 sccm, 1 sccm, and 1.5 sccm) were used. In addition, 0.5 sccm oxygen precursor at other temperatures of 1300 °C and 1400 °C were also used to investigate the structure evolution. Top view and side view scanning electron microscopy (SEM) images of the nanowires were taken directly from the as-synthesized substrates. To obtain transmission electron microscopy (TEM) of nanowires, they were transferred to the TEM grid using the conventional alcohol dispersion and transference technology. The supersaturation of the system was defined as following^20^:

1 $$\sigma =\frac{{P}}{{{P}}_{{\rm{e}}}}-1$$

where *P* is the partial pressure of oxygen, *P*_*e*_ is the equilibrium partial pressure of oxygen at the deposition location. Details of the calculation process of *P* and *P*_*e*_ were reported elsewhere (ref. 7).

## Results and Discussion

**Figure 1:**
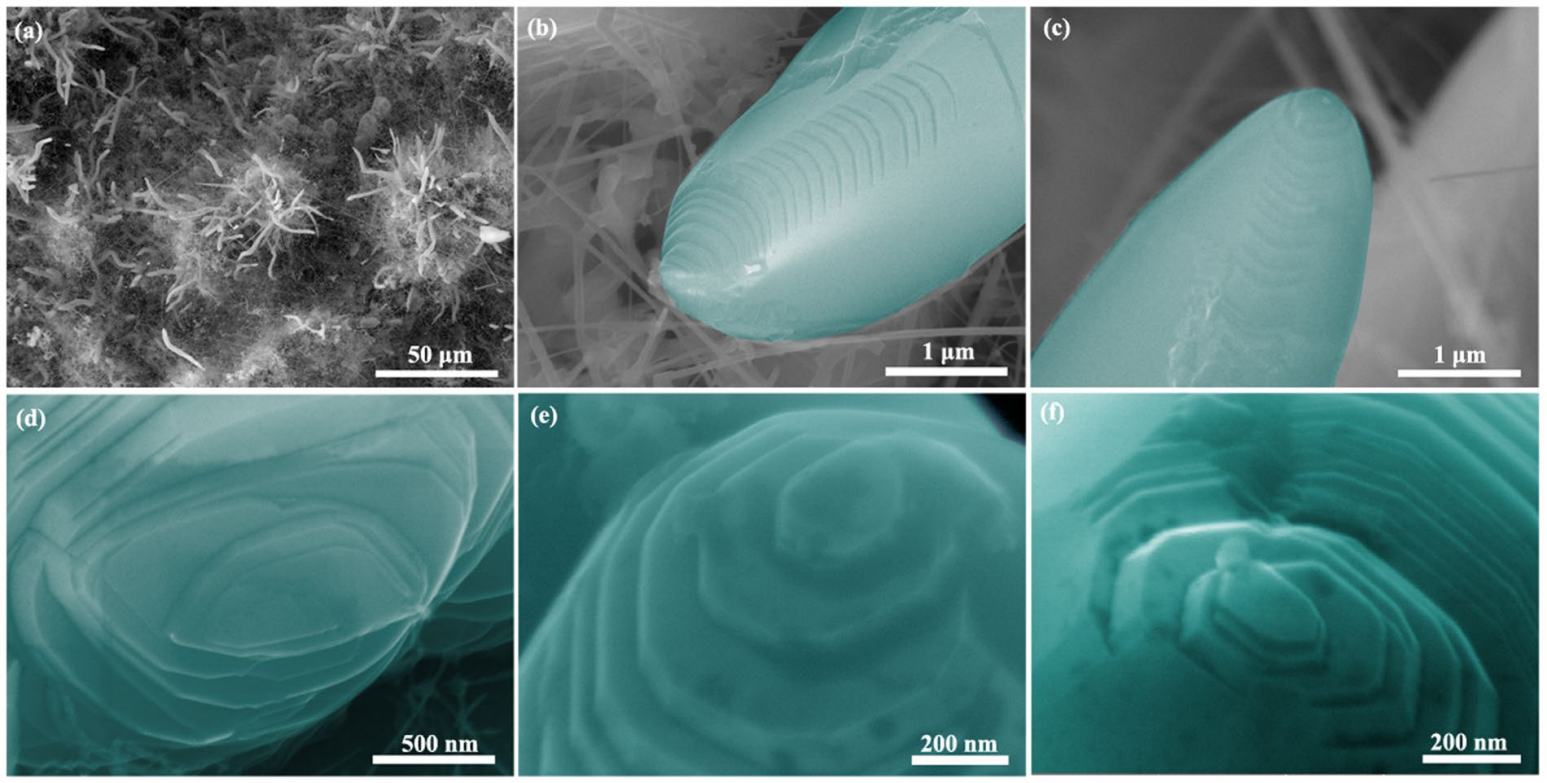
(**a**) SEM surface image of the as-synthesized α-Al_2_O_3_ nanowires on the as-prepared SiC-Si-Al_2_O_3_ ceramics; (**b**) and (**c**) SEM images of a representative screw dislocation core on the conical tip of﻿ α-Al_2_O_3_ nanowires; (**d**), (**e**) and (**f**) high-magnification SEM images of the representative screw dislocation core on the tip of α-Al_2_O_3_ nanowires.

The morphology of the products on the as-prepared SiC-Si-Al_2_O_3_ ceramics is investigated by SEM. The low-magnification SEM image (Fig. 1a) indicates that the products consist of a large quantity of the nanowires with the diameter of 1–3 μm. Their lengths are up to several tens of micrometers. A thread-like morphology is clearly observed along one of the sides of the nanowires, as shown in Fig. 1b and c. All of the nanowires possess the conical tip, on which a step terminates within a flat terrace. The high-magnification SEM images are also taken from the direction down on the nanowire tip and along its axis direction, as shown in Fig. 1d,e and d. From Fig. 1d, it can be seen that the nanowire contains a single helical pattern and a helical core located at the center. In general, it possesses the morphology similar to hillocks, each basal plane has an oval shape and gradually shrinks to the center summit layer by layer when spiraling up. Figure 1e shows that the nanowire presents a stepwise spiral terrace configuration on their top surface with their diameters decreasing continuously. An interesting phenomenon is that the terraces are evolved initially at the edge and then spread out to generate the flat top terrace along a spiral center, as shown in Fig. 1f. It clearly demonstrates the spiral step evolution path. These surface features fit well to a screw dislocation growth pattern, since a spiral center is observed that is the characteristic feature of a screw dislocation-driven growth.

**Figure 2:**
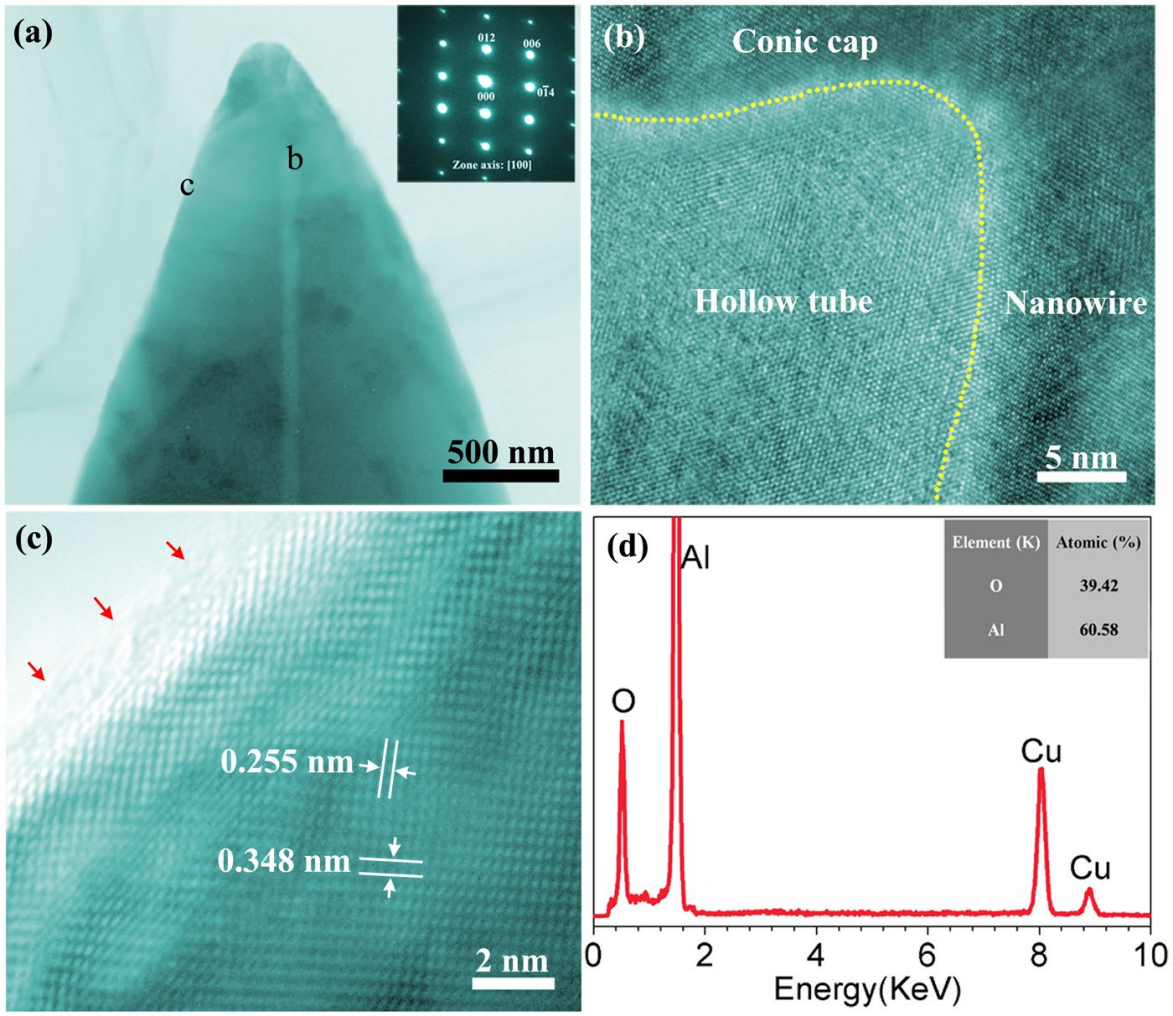
(**a**) TEM image of the individual α-Al_2_O_3_ nanowire with a conical tip (The inset is the corresponding SAED pattern); (**b**) HRTEM image of the areas marked as “b”; (**c**) HRTEM image of the areas marked as “c”; (**d**) EDS spectrum obtained from the individual α-Al_2_O_3_ nanowire (The inset is the ratio of O and Al atoms).

More details associated to the morphology and crystal structure of the as-synthesized nanowires are investigated using TEM. Figure 2a shows typical TEM image of the individual nanowire with a conical tip, where a narrow hollow tube structure is observed. This hollow tube structure is not expected to be generated within the whole nanowire along its length directions, since it is not observed within the top of the conical tip (Fig. 2b). This is in agreement to the SEM observations, where no pipe is detect on the surface of the nanowire top. It is also worth noticing that the length of the solid conical tip is short as only several hundreds of nanometers, as shown in Fig. 2a. In brief, the as-synthesized nanowires possess a solid conical cap-closing hollow tube structure. The selected area electron diffraction (SAED) pattern of the individual nanowire (top-right corner in Fig. 2a) along zone axis of [100] indicates that the nanowires are the typical single crystal of α-Al_2_O_3_. The high resolution TEM (HRTEM) image of the individual nanowire in Fig. 2c shows a periodic lattice structure and two sets of fringes with the d-spaces of 0.255 nm and 0.348 nm, corresponding to the (104) and (012) planes of α-Al_2_O_3_ (JCPDS Card No. 10–0173), respectively. Both HRTEM image and SAED pattern suggest that the as-synthesized nanowires are single-crystalline α-Al_2_O_3_. The energy dispersive X-ray spectroscopy (EDS) spectrum (Fig. 2d) indicates that the nanowires mainly contain Al and O elements (Cu element comes from the copper grid used to support TEM sample). The atomic ratio of Al to O of the nanowires is calculated as 2:3, which confirms the composition of the nanowires as Al_2_O_3._ Moreover, HRTEM image also displays an amorphous layer of ~1 nm on the nanowire surface (indicated by red arrows in Fig. 2c). Combining this to the Al and O elements detected by EDS spectrum (see Fig. 2d) indicates that there is an amorphous Al_2_O_3_ layer on the nanowire surface.

The strain energy imbalance related to a coaxial screw dislocation in a cylinder also can result in a torque around the dislocation known as the Eshelby twist, which has been observed and confirmed in some nanowires and nanotubes of ZnO, PbS and PbSe^6, 15, 18^. In the present work, the structure of the as-synthesized α-Al_2_O_3_ nanowires consists of the hollow tube and the solid conic cap. For a hollow cylinder, the Eshelby twist can be mathematically expressed as^18^:

2 $${\alpha }=\frac{{b}}{{\pi }{{R}}^{{2}}+{\pi }{{r}}^{{2}}}$$

In contrast, for a solid cylinder, the Eshelby twist can be written as^15^:

3 $${\alpha }=\frac{{b}}{\pi {{R}}^{2}}$$

where *α* is the lattice twist in radians per unit length, *b* is the Burgers vector, *R* is the radius of the cylinder, *r* is the radius of the hollow tube. Although Eshelby twist in 1D nanomaterials can be observed using several common microscopy or diffraction techniques, the twist must be sufficiently dramatic for easy observation. Especially for the micrometer-size crystals, the Eshelby twist is hard to be observed, since a 1/(*R* ^*2* +^ *r* ^*2*^) or 1/*R* ^*2*^ dependence makes the twist less significant at the micrometer-size compared to the nanoscale. Thus, although we attempt to observe the Eshelby twist in the as-synthesized α-Al_2_O_3_ nanowires, the results are inconclusive.

**Figure 3:**
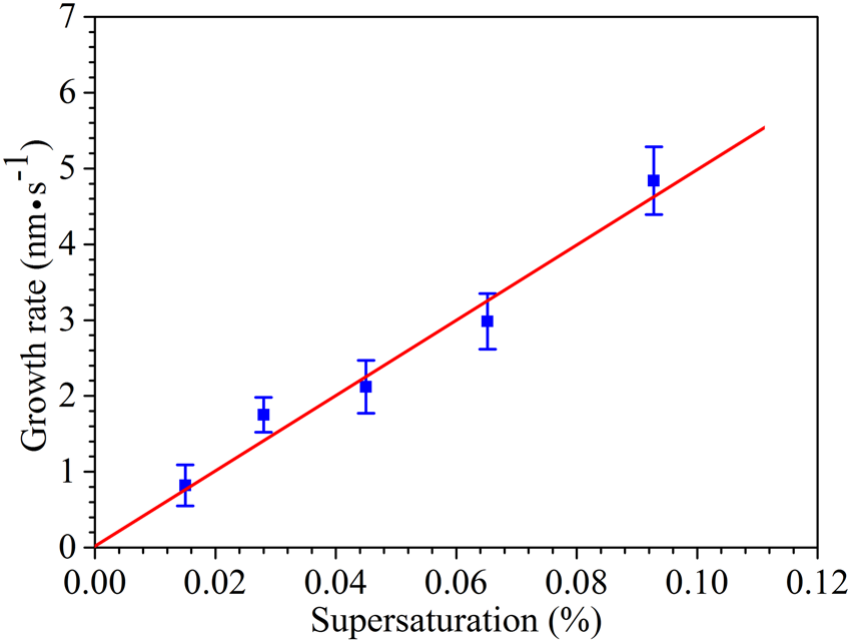
The fitted curve of the growth rates for α-Al_2_O_3_ nanowires as a function of supersaturation.

In order to further demonstrate that the growth of the as-synthesized α-Al_2_O_3_ nanowires is driven by axial screw dislocations, we compare experimentally determined growth kinetics with those predicted using fundamental Burton-Cabrera-Frank (BCF) crystal growth theory. Briefly, BCF theory predicts a dislocation-growth rate (*R*_*dislocation*_) linearly dependent on supersaturation (*σ*)^18^.

4 $${{R}}_{{dislocation}}=C{\sigma }$$

where *C* is the rate constant. We have determined experimental axial growth rates by conducting a series of growth experiments over a range of oxygen flow rates from 0.5 sccm to 2 sccm and surveying the lengths of α-Al_2_O_3_ nanowires. These experimental growth rates are plotted as a function of supersaturation, as shown in Fig. 3. It is clear that the experimentally measured growth rates of α-Al_2_O_3_ nanowires follow the rate law predicted by dislocation-driven growth, which strongly supporting the assertion that the as-synthesized α-Al_2_O_3_ nanowires grow via the screw dislocation-driven growth mechanism.

In general, the screw dislocation-driven growth of α-Al_2_O_3_ nanowires with the solid conical cap-closing hollow tube structure can be divided into two stages, that is the dislocation-driven growth of the hollow tube structure and the dislocation-driven growth of the solid conical cap structure. The strain energy associated with screw dislocations originating from the disruption of the perfect periodicity within the crystal lattice plays a crucial role in their growth process. For a hollow cylinder, the strain energy per unit length (*E*) can be expressed as^21^:

5 $${E}=\frac{{{b}}^{{2}}{\mu }}{4{\pi }}\,\mathrm{ln}(\frac{{R}}{{r}})$$

In contrast, for a solid cylinder, the strain energy per unit length can be expressed as^6^:

6 $${E}=\frac{{{b}}^{{2}}{\mu }}{4{\pi }}\,\mathrm{ln}(\frac{{R}}{{{r}}_{{0}}})$$

where *µ* is the shear modulus, *r*_*0*_ is the core radius of the dislocation. With an increasing magnitude of *b*, the strain energy within the crystal will enhance until exceeding eventually the surface energy required for creating a new inner surface and cause the dislocation core to become hollow. The radius of this hollow at the equilibrium is directly dependent on the magnitude of *b* ^21^:

7 $${r}=\frac{{{b}}^{{2}}{\mu }}{8{{\pi }}^{{2}}{\gamma }}$$

where *γ* is the surface energy. Therefore, when the *b* is sufficiently large, the equilibrium structure of dislocation-driven nanowires is expected to be a hollow structure. Such mechanism has been widely explained the frequently observed micropipe structures in dislocation-prone materials such as SiC and GaN^22, 23^. In the present work, the radius of hollow tube structure of the nanowires is considered to be large (Fig. 2a). Therefore, it requires a large magnitude of *b* to maintain a large strain energy, and further drives the regular screw dislocation growing into a hollow tube with the self-perpetuating steps driving elongation. In the later stage, the altered growth environment changes the strain energy and breaks the energy balance. This results in a small magnitude of b and closes up the hollow tube growth. Therefore, the afterwards growth of the hollow tube structure ends up and turns into the growth of the solid conical cap structure by axial screw dislocations. In particular, the final lengths of the solid conical cap structures in the as-synthesized α-Al_2_O_3_ nanowires are short as only several hundreds of nanometers, as shown in Fig. 2a. Summarizing the above-mentioned facts indicate the possibility that the solid conical cap structure may be generated in the cooling process.

**Figure 4:**
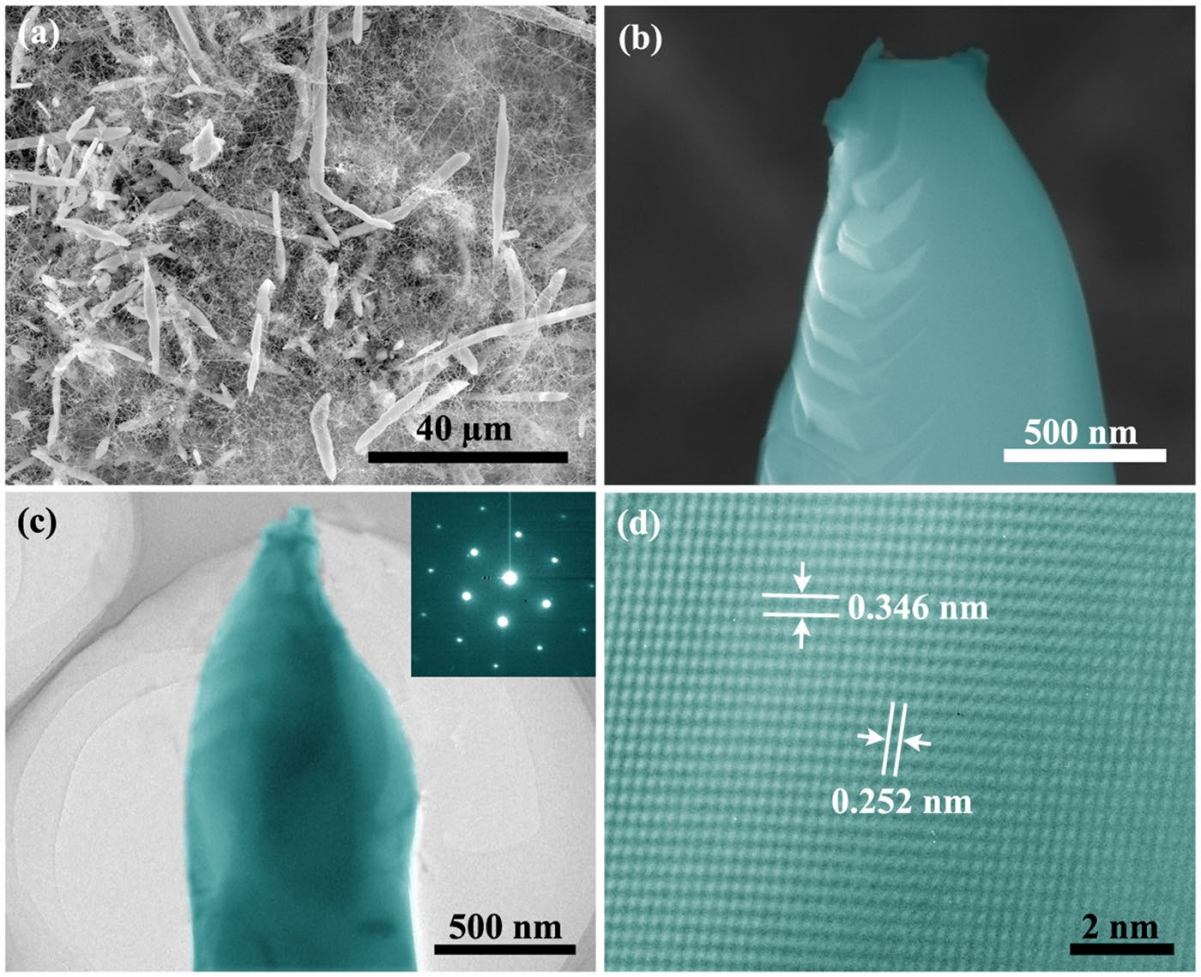
(**a**) SEM surface image of α-Al_2_O_3_ nanowires on the as-prepared SiC-Si-Al_2_O_3_ ceramics synthesized at 1400 °C with oxygen flow rate of 0.5 sccm; (**b**) SEM image of a representative screw dislocation core on the conical tip﻿ of α-Al_2_O_3_ nanowires; (**c**) TEM image of the individual Al_2_O_3_ nanowire with a conical tip (The inset is the corresponding SAED pattern); (**b**) HRTEM image of the individual α-Al_2_O_3_ nanowire.

To verify above understanding and build a simulation environment for the growth of solid conical cap of the nanowires in the cooling process, the deposition temperatures are changed to 1400 °C and 1300 °C under lower supersaturation (the flow rate of oxygen gas is only 0.5 sccm). In those situations, Al_2_O_3_ nanowires are not present at 1300 °C, but are synthesized at 1400 °C. It is worth noticing that the morphology of these nanowires synthesized at 1400 °C is in agreement to the nanowires synthesized at 1500 °C, as shown in Fig. 4a. The nanowires are several ten micrometers long with diameters of 1–3 μm. Each of them has a terraced conical tip on which the central terrace exhibits a flat surface and the existence of screw dislocation can be observed by SEM characterizations (Fig. 4b). These observations confirm that the screw dislocation-driven growth mechanism in the formation of these nanowires. Moreover, the average step height of these nanowires measured for 50 individual nanowires is about 81 nm, which is less than the ones with the solid conical cap-closing hollow tube structure (23 nm). According to BCF theory, the step height (*h*) is quadratically dependent on the magnitude of *b* ^21^

8 $${h}=\frac{{8}{{\pi }}^{{2}}{r}{{\text{'}}}^{{2}}{\gamma }{\text{'}}}{{\rho }{{b}}^{{2}}{\mu }}$$

where *γ*′ is the specific free energy per unit length of a step, *1/ρ* is the equilibrium curvature, *r*′ is *r* for the hollow tube or *r*_*0*_ for the solid cylinder. From Eq. 8, it can be concluded that the magnitude of *b* in these screw dislocation-drive growth nanowires is less than that in the nanowires with the solid conical cap-closing hollow tube structure. This implies that these nanowires are not hollow tube but solid structure. TEM characterizations show that these nanowires with the conical tip is indeed a solid structure rather than hollow tube structure, as shown in Fig. 4c. SAED pattern (top-right corner in Fig. 4c) indicates that these nanowires possess a typical single crystal structure. HRTEM image (Fig. 4d) shows that these nanowires possess a periodic lattice structure with two sets of fringes with the d-spaces of 0.252 nm and 0.346 nm, corresponding to the (104) and (012) planes of α-Al_2_O_3_ (JCPDS Card No. 10-0173), respectively. Both HRTEM image and SAED pattern indicate that these nanowires are single-crystalline α-Al_2_O_3_. These features suggest that the growth mode of these nanowires is the screw dislocation-driven growth. However, the changes in the growth environment induce the structure transition of these nanowires. This demonstrates that the solid conical cap is generated in the cooling process for the nanowires with the solid conical cap-closing hollow tube structure.

**Figure 5:**
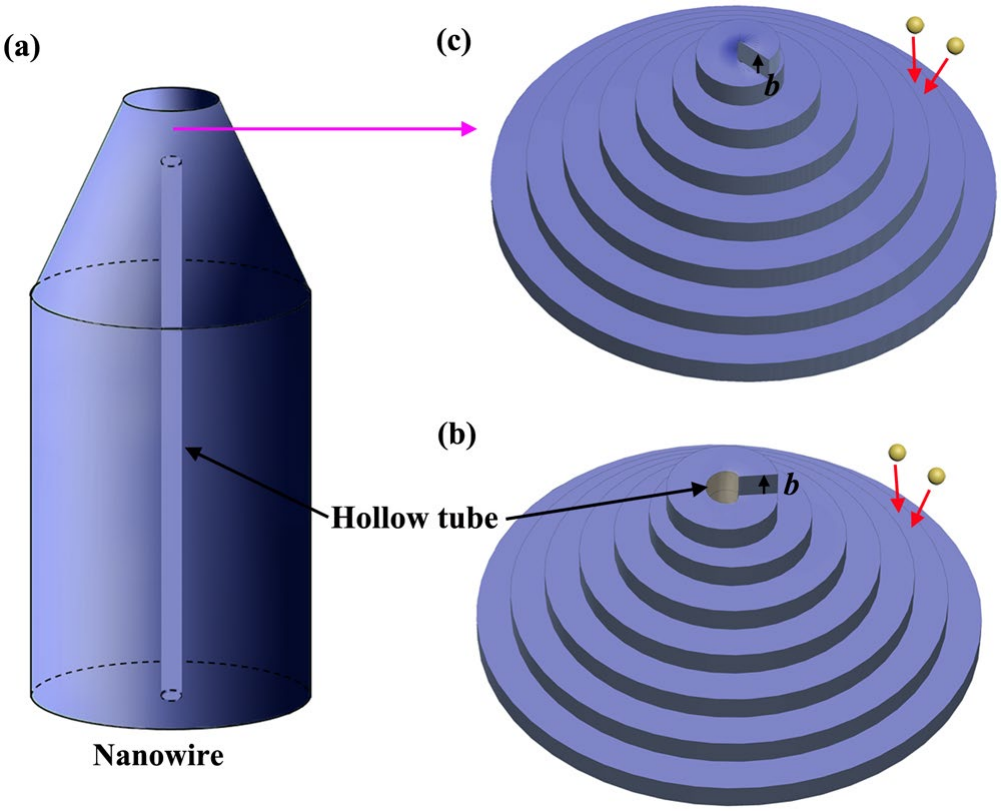
Schematic diagram of the growth process for α-Al_2_O_3_ nanowires. (**a**) The structure of α-Al_2_O_3_ nanowires; (**b**) the formation of the hollow tube structure in α-Al_2_O_3_ nanowires by axial screw dislocations, where the *b* is shown; (**c**) the formation of the solid structure in α-Al_2_O_3_ nanowires by axial screw dislocations, where the *b* is shown.

The screw dislocation-driven growth has been demonstrated to be a generalized mechanism for the growth of the crystals, which occurs when the supersaturation of system is relatively low. When only a small amount of oxygen is used in the system, the as-synthesized α-Al_2_O_3_ nanowires is expected to follow a screw dislocation-driven growth mode. However, unlike the reported screw dislocation-driven growth of the nanowires or nanotubes, the as-synthesized α-Al_2_O_3_ nanowires in the present work shows a solid conical cap-closing hollow tube structure with the screw dislocations. Therefore, it indicates a new solid conical cap-closing hollow tube growth model by axial screw dislocations for α-Al_2_O_3_ nanowires. A schematic diagram of the possible growth process of the as-synthesized α-Al_2_O_3_ nanowires was presented in Fig. 5. Ideally, the introduced oxygen will first react with the ceramic substrate to generate the gaseous products under high-temperature conditions. Afterwards, the resulting gaseous species react and nucleate on the substrates and grow up to α-Al_2_O_3_ crystals. At the same time, a screw dislocation can be generated through an accidental overgrowth of two exposed edges at the initial stage due to the formation of large amount of α-Al_2_O_3_ within a short time, leading to a slipped edge as a screw defect. Based on BCF theory, once the screw dislocation is created, the crystal growth at the edge of the dislocation is energetically favorable, which will result in a spiral growth of the crystal. Under the grown environment, the crystal contains strain energy associated with screw dislocations. The energy exceeds the one required for creating a new inner surface to form 1D nanowire crystal structures with hollow core, in which case the axial screw dislocations provide self-perpetuating steps to enable the nanowire growth, as shown in Fig. 5b. By increasing the growth time, the nanowires with hollow tube structure are formed, as shown in Fig. 5a. Similar structures have also been demonstrated previously in other oxide nanowires, such as In_2_O^24^ and Cu_2_O^25^. However, in the cooling process, the strain energy associated with screw dislocations is expected to be decreased owning to the changes in the growth environment of the crystal. When this strain energy is less than the energy required for creating a new inner surface, the screw dislocation-driven growth of the hollow tube structure within the nanowires will be terminated and transformed to a screw dislocation-driven growth of the solid structure, as shown in Fig. 5c. A solid structure is formed by the screw dislocations in the growth of the nanowires. In that case, the self-perpetuating steps originating from the screw dislocations enable the nanowire growth to form a solid conical cap on the top of the nanowires (Fig. 5a). The proposed solid conical cap-closing hollow tube growth model, based on the axial screw dislocations, is expected to be a generalized growth mechanism for nanowires within low supersaturation.

## Conclusions

We demonstrate a solid conical cap-closing hollow tube growth model by axial screw dislocations in nanowire formation from vapor phase. In the first step, the axial screw dislocations in the nanowires result in the spontaneous formation of hollow tube structure as the equilibrium morphology. Afterwards, such hollow tube is closed up to form a solid conical cap on the nanowire top by axial screw dislocations due to the altered the growth conditions. The terrace, hollow tube and solid features on (or within) these nanowires provide the possibility to further understand the solid conical cap-closing hollow tube growth kinetics by axial screw dislocations. The growth kinetics of the nanowires with such structure is in agreement to the predicted dislocation growth mechanism by using BCF theory.
